# Structure-Based Prediction of Asparagine and Aspartate Degradation Sites in Antibody Variable Regions

**DOI:** 10.1371/journal.pone.0100736

**Published:** 2014-06-24

**Authors:** Jasmin F. Sydow, Florian Lipsmeier, Vincent Larraillet, Maximiliane Hilger, Bjoern Mautz, Michael Mølhøj, Jan Kuentzer, Stefan Klostermann, Juergen Schoch, Hans R. Voelger, Joerg T. Regula, Patrick Cramer, Apollon Papadimitriou, Hubert Kettenberger

**Affiliations:** 1 Large Molecule Research, Roche Pharmaceutical Research and Early Development, Penzberg, Germany; 2 Disease & Translational Informatics, Roche Pharmaceutical Research and Early Development, Penzberg, Germany; 3 Therapeutics Modalities Informatics, Roche Pharmaceutical Research and Early Development, Penzberg, Germany; 4 Pharmaceutical Sciences, Roche Pharmaceutical Research and Early Development, Penzberg, Germany; 5 Gene Center Munich and Department of Biochemistry, Center for Integrated Protein Science (CIPSM), Ludwig-Maximilians-Universität (LMU), Munich, Germany; Technical University of Braunschweig, Germany

## Abstract

Monoclonal antibodies (mAbs) and proteins containing antibody domains are the most prevalent class of biotherapeutics in diverse indication areas. Today, established techniques such as immunization or phage display allow for an efficient generation of new mAbs. Besides functional properties, the stability of future therapeutic mAbs is a key selection criterion which is essential for the development of a drug candidate into a marketed product. Therapeutic proteins may degrade via asparagine (Asn) deamidation and aspartate (Asp) isomerization, but the factors responsible for such degradation remain poorly understood. We studied the structural properties of a large, uniform dataset of Asn and Asp residues in the variable domains of antibodies. Their structural parameters were correlated with the degradation propensities measured by mass spectrometry. We show that degradation hotspots can be characterized by their conformational flexibility, the size of the C-terminally flanking amino acid residue, and secondary structural parameters. From these results we derive an accurate *in silico* prediction method for the degradation propensity of both Asn and Asp residues in the complementarity-determining regions (CDRs) of mAbs.

## Introduction

Monoclonal antibodies (mAbs) and new antibody domain-based molecules constitute the majority of protein therapeutics under clinical investigation [1], [2] for severe malignancies such as cancer, viral and inflammatory diseases. mAbs are potent in a diverse range of therapeutic indications, and are readily generated against promising new targets. The specificity of mAbs is determined by sequences in the CDRs located in the variable F_v_ domain. The process of selecting the clinical candidate mAb typically starts with large-scale screening for functional properties. Screening is followed by detailed *in vitro* profiling of multiple mAbs to identify candidates that fulfill all desired functional criteria. To ensure optimal technical development and *in vivo* stability, potentially instable mAbs have to be identified and excluded during the lead selection process.

During manufacturing, storage and *in vivo*, therapeutic antibodies are at risk for degradation via a number of pathways (reviewed by [3]). Amongst the most frequently occurring degradation reactions in proteins are the chemical degradation of Asn [4] and Asp residues [5], [6]. While these reactions may be kept under control by appropriate storage and formulation conditions [7]–[10] of the final drug substance and drug product, degradation during fermentation, downstream-processing, and *in vivo* can often not be controlled sufficiently. If Asn and Asp residues are involved in antigen recognition, their chemical alteration can lead to severe loss of potency [11]–[15]. In several cases, these degradation events were reported to hamper long-term mAb functionality [11], [12], [14], [16]–[19]. *In vivo*, protein degradation events are described in connection with protein ageing [20]–[26], with cancer by triggering apoptosis [27]–[29] or with severe effects on other biological functions, e. g. stability decrease of human lens betaA3-crystallin, abnormal MAPK signaling, the alteration of potential beta-secretase efficacy and specificity in the course of Abeta generation, or increase of lysozyme lytic activity against bacterial cells [30]–[37]. The identification of degradation-prone drug candidates is ideally done early in the drug development process to either adjust the manufacturing and formulation process accordingly or to re-engineer a problematic candidate to remove such hotspots [38].

**Figure 1:**
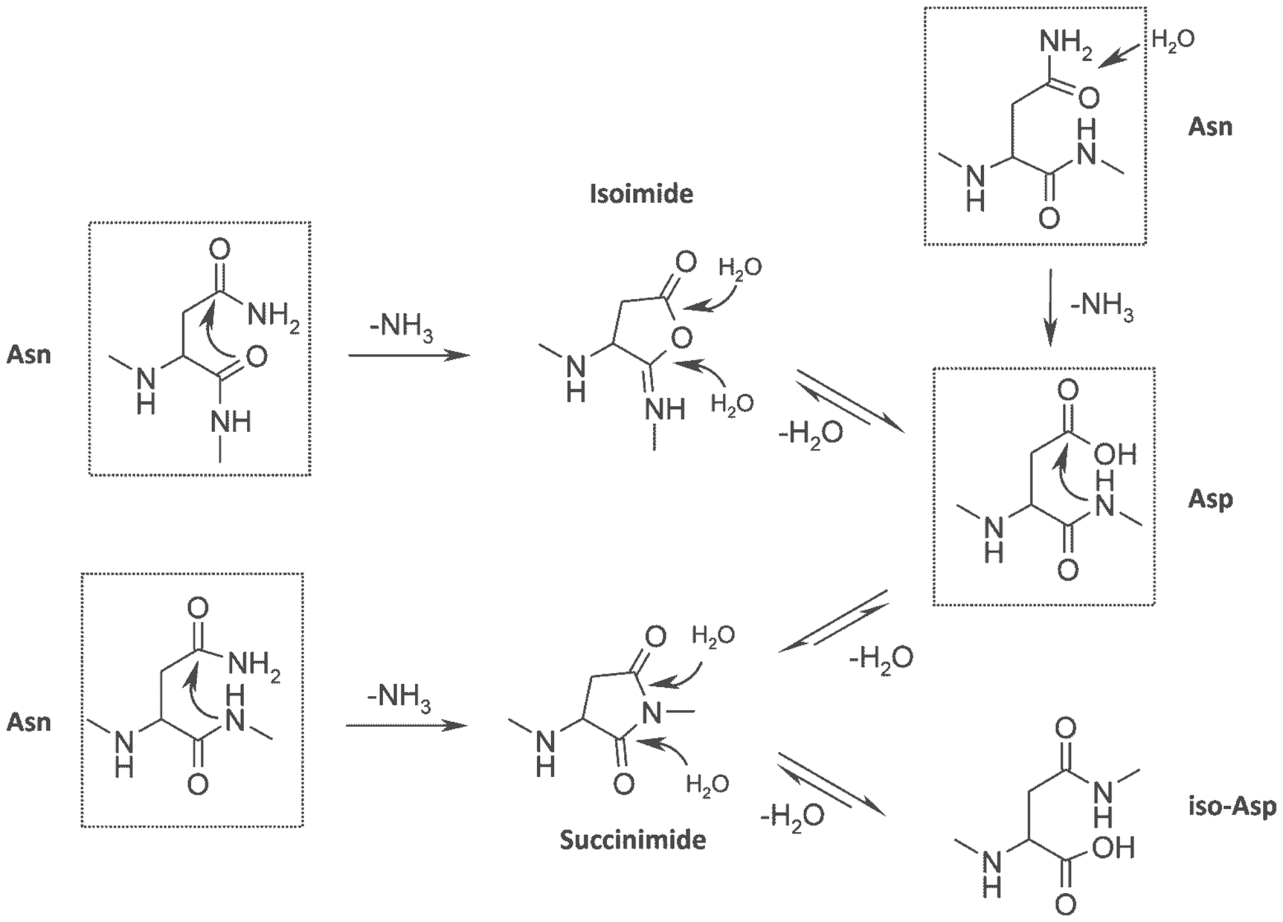
Asparagine and Aspartate degradation pathways. Deamidation of asparagine or dehydration of aspartic acid occurs by nucleophilic attack of the α-amino group of the C-terminally flanking amino acid. This leads to formation of a metastable succinimide (cyclic imide) intermediate, which hydrolyzes to a mixture of aspartyl and iso-aspartyl linkages. Alternatively, nucleophilic attack by the backbone carbonyl oxygen results in a cyclic isoimide intermediate, yielding only aspartyl residues after hydrolysis independent of the point of attack of the incoming water molecule. Asparagine residues can deamidate to Asp by direct water-assisted hydrolysis. Standard amino acids (Asn, Asp) are outlined with black boxes.

Asn and Asp residues share a degradation pathway that proceeds via the formation of a cyclic succinimide intermediate (Figure 1) [4], [6], [39]. Succinimide results from deamidation of Asn or dehydration of Asp by nucleophilic attack of the backbone nitrogen of the succeeding amino acid on the Asn/Asp side chain γ-carbonyl group. The metastable cyclic imide can hydrolyze at either one of its two carbonyl groups to form aspartyl or iso-aspartyl linkages in different ratios, depending on hydrolysis conditions and conformational restraints [4], [6], [18], [25], [40], [41]. In addition, alternative degradation mechanisms for Asn were proposed [11] such as nucleophilic attack by the backbone carbonyl oxygen to form a cyclic isoimide [6], [42], [43] or direct hydrolysis of Asn to Asp [44], [45] (Figure 1). Several analytical methods, mostly charge-sensitive methods such as ion exchange chromatography or isoelectric focusing, were described to detect either of the degradation products, i.e. succinimide, Asp or isoAsp [14], [46]–[48]. Most suitable for the quantification and the localization of degradation sites in proteins is the analysis via liquid chromatography tandem mass spectrometry (LC-MS/MS) [13], [14], [16], [19], [43], [49]–[56].

Diverse parameters were proposed which may influence the degradation propensity of Asn and Asp residues, e.g. the primary sequence [4], [6], [21], [39], [45], [57]–[62], the solvent dielectric constant, temperature, and the pH, mostly in the peptide [58], [59], [63]–[65], but also in the protein context [8], [11], [18], [66]. Already in the 1980s, several structural requirements were suggested as principal determinants for protein deamidation [6], [67] which have later been confirmed and extended [16], [40], [42], [43], [45], [57], [68]–[70].

Despite accumulated knowledge about the degradation mechanism and its structural requirements, the reliable prediction of deamidation and isomerization in mAbs remains an unresolved issue. In the early stages of drug discovery, the amino acid sequence is often known for a large number of lead candidates, but the protein amounts available for *in vitro* stability testing are often limited and the necessary mass spectrometry assays are labor intensive and time consuming. Thus, the possibility to reliably predict Asp and Asn hotspots without the need for experiments is key to the rapid identification of stable F_v_ sequences early in the discovery phase.

To shed light on the complex interplay of several parameters potentially leading to chemical degradation, we generated a uniform experimental data set of site-specific degradation events before and after “stress” treatment in 37 mAbs by mass spectrometry. These *in vitro* data combined with structural parameters derived from homology models were used to study the quantitative contribution of structural parameters in the degradation pathway, and to develop an *in silico* approach for the identification and selection of chemically stable mAbs during the clinical candidate generation process.

## Results

### Experimental survey of antibody degradation sites and rates

In order to determine the driving factors for Asn and Asp degradation sites in the F_v_ regions of mAbs, analytical, structural, and computational methods were combined. A collection of 37 different therapeutic IgG1, IgG2 and IgG4 mAbs (in-house as well as marketed products) was investigated (Table 1, Materials and Methods). These antibodies were subjected to forced degradation (“stress”) at a typical formulation pH of 6.0 at 40°C for 2 weeks (Material and Methods), and subsequently analyzed for degradation events by mass spectrometric analysis after tryptic digestion. Thereby the affected residues were identified and the amount of modification in stressed and corresponding reference samples was quantified (Materials and Methods). Modifications already present in unstressed samples, for instance due to poor stability at physiological pH during fermentation or induced during bioprocessing, were also detected. To avoid further modification and to stabilize the cyclic imide intermediate, the pH was maintained at 6.0 during peptide map sample preparation [54], [71]. The evaluation of the entire set of 74 LC-MS/MS peptide mapping experiments from 37 stressed and corresponding reference samples enabled us to detect all possible products of Asn and Asp degradation, i.e. the succinimide intermediate, iso-Asp, and Asp (example in Figure S1). Out of all 559 Asn and Asp residues in the F_v_ regions of the 37 mAbs, 60 residues (11%) exhibit quantifiable amounts of modification. We sub-classified these into 21 hotspots (Table 1), 14 weak spots (Table S1), and 24 reactive spots (Table S2). The term hotspot corresponds to ≥3%, weak spot to ≥1 and <3%, and reactive spot to <1% modification in the stressed samples. In the data set used for statistical evaluation, only hotspots and non-hotspots were considered. In order to achieve a reliable, unambiguous dataset, reactive spots and weak spots, as well as hotspots with unclear assignment or within an F_v_ N-glycosylation site were excluded from the dataset.

**Table 1:** Experimental Asn and Asp hotspot collection.

mAb	modifi-cation	% modified (stressed)	% modified (un-stressed)	motif	location
mAb22	iD+suc	39	14	DG	HC CDR 3
Omalizumab [12]	iD+suc	31	3	DG	LC CDR 1
mAb2	iD+**suc**	26	3	DS	LC CDR 2
Trastuzumab [11], [54]	**dea** *+suc	24	11	NT	LC CDR 1
Trastuzumab [11], [54]	**iD**+suc	22	7	DG	HC CDR 3
mAb14	dea	22	n.a.	NS	LC CDR 3
mAb1‡	dea+suc	17	5	NT	HC CDR 3
mAb22	iD+suc	12	6	DG	LC CDR 2
mAb13	iD+suc	10	n.a.	DG	HC CDR 3
Nimotuzumab	iD+suc	9	2	DS	HC CDR 3
mAb26	dea	8	3	NG	LC CDR 1
Nimotuzumab‡	dea# **+suc** #	8	5	?	LC CDR 1
mAb32	dea	6	5	NS	HC CDR 2
Infliximab	dea	6	2	NS	HC CDR 2
Natalizumab	dea+suc	5	3	NG	HC CDR 2
Trastuzumab [11], [54]	dea+suc	5	4	NG	HC CDR 2
mAb17	**dea**+suc	4	1	NS	LC CDR 1
mAb14	suc	4	0	NN	LC CDR 1
mAb11	suc	4	2	NT	LC CDR 1
mab48	dea+suc	3	2	NG	HC CDR 2
mAb2	iD+suc	3	n.a.	DS	HC CDR 3

*only Asp as deamidation species.

‡ excluded from hotspot data set because of interaction with a CDR glycosylation site which is not represented by the homology models.

# proof of modification site impossible with available methods (tryptic peptide, AspN peptide, CID fragmentation, HCD fragmentation), thus excluded from the hotspot data set.

Main modifications are written in bold. iD = isomerization, suc = succinimide, dea = deamidation, n.a.: not assessed.

### Degradation sites are exclusively located in CDRs

Strikingly, all degradation hotspots are located in the CDR loops (Table 1). Thus, the C_H_1/C_L_ domains and the F_v_ framework represent a stable scaffold. Most hotspots are located in the light chain CDR 1 and the heavy chain CDR 3, whereas in our dataset heavy chain CDR 1 does not contain any hotspot. In summary, 15 out of 37 analyzed mAbs contain at least one Asn or Asp hotspot in one of the CDRs.

**Figure 2:**
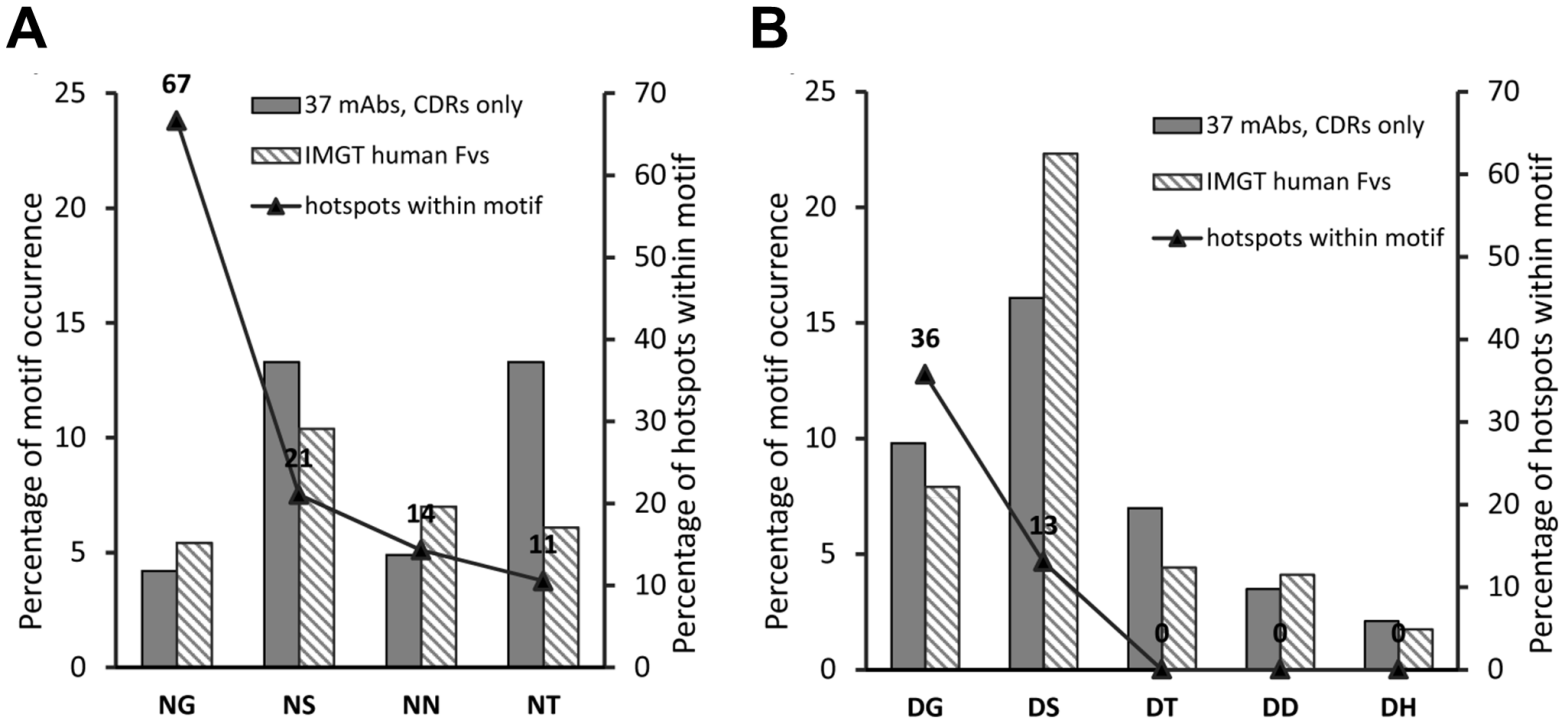
Occurrence of Asn and Asp amino acid motifs in the CDRs of a therapeutic mAb collection and a set of naturally occurring antibodies (IMGT). Black triangles show percentages of hotspots within Asn and Asp motifs of the experimental collection of 37 mAbs. Bars represent percentages of depicted sequence motifs among all Asn or Asp residues in only CDR regions. Percentages shown as filled bars represent the non-redundant collection of the 37 analytically assessed therapeutic mAbs, bars striped in light grey belong to a collection of 9990 V-D-J- and 6296 V-J regions of naturally occurring antibodies from the IMGT database. (**A**) Asn sequence motifs, (**B**) Asp sequence motifs.

It was shown in previous studies that the amino acid residue succeeding Asn and Asp influences the rate of succinimide formation in proteins [45], [57]. So far, eight different sequence motifs involved in chemical degradation within F_v_ regions of therapeutic antibodies have been described (Asn succeeded by Gly, Ser, or Thr, and Asp succeeded by Gly, Ser, Thr, Asp, or His) [11]–[18], [72]–[79]. In accordance with previous observations, Asn-Gly and Asp-Gly motifs are by far the motifs most prone to modification in our data set, accounting for 67 and 36% of hotspots observed, respectively (Figure 2). All described sequence motifs except Asp-Thr and Asp-His were observed as degradation sites in the CDRs of our antibody collection. In addition, chemical degradation was detected at an Asn-Asn motif in mAb14 (Table 1). Degradation at this sequence motif has so far not been described in antibody CDRs, but in other proteins [45].

To assess the relevance of our therapeutic mAb collection in relation to naturally occurring antibodies, the frequency of the known Asn and Asp degradation sequence motifs (NG, NN, NS, NT, DG, DS, DT, DD, DH) was compared between the CDRs of our mAb collection (combined Kabat and Chothia definitions [80]) and 16286 naturally occurring human mAb sequences (9990 V-D-J and 6296 V-J sequences) from the international ImMunoGeneTics (IMGT) information system's monoclonal antibody database (www.IMGT.org). Despite the enormous difference in size of the compared datasets, the frequency at which Asn and Asp motifs occur, is distributed comparatively equally and shows that the sequence composition of the investigated antibody molecules contains low bias (Figure 2). The only exception is the NT motif that is found twice as frequently in therapeutic mAbs than in IMGT. Obviously, the most degradation-prone Asn-Gly and Asp-Gly motifs are comparatively infrequent.

### Analysis of degradation site structure

The structural environment of Asn and Asp hotspots and non-hotspots in the antibodies' F_v_ fragments was characterized by a set of 20 parameters with a putative role in the degradation mechanism. Homology models of F_v_ fragments were generated by a state-of-the art homology modeling software (Materials and Methods). Parameters were extracted from these homology models by an automated procedure (Materials and Methods). Generally, the high homology to template structures typically results in precise homology models of framework and short CDR regions. However, modeling of long CDR loops is prone to large modeling uncertainties, possibly due to the high inherent flexibility of such loops [81]–[84]. Therefore, all CDRs were subjected to an additional loop modeling procedure [85] (Materials and Methods), yielding a five-membered homology model ensemble. Like this, additional information on different possible CDR conformations was captured (Figure S2), without the necessity of computationally demanding molecular dynamics simulations. Moreover, bias in homology models generated from templates with bound antigen is removed by the loop refinement procedure which models loops using experimental loop structures from a loop database, followed by energy minimization. The correlation between structural parameters and *in vitro* degradation was investigated by machine-learning algorithms. Statistical validation of the predicting model shows promising accuracy and low mis-prediction compared to sequence motif-based prediction.

### A set of 20 parameters describes the structural environment of Asn and Asp residues

**Figure 3:**
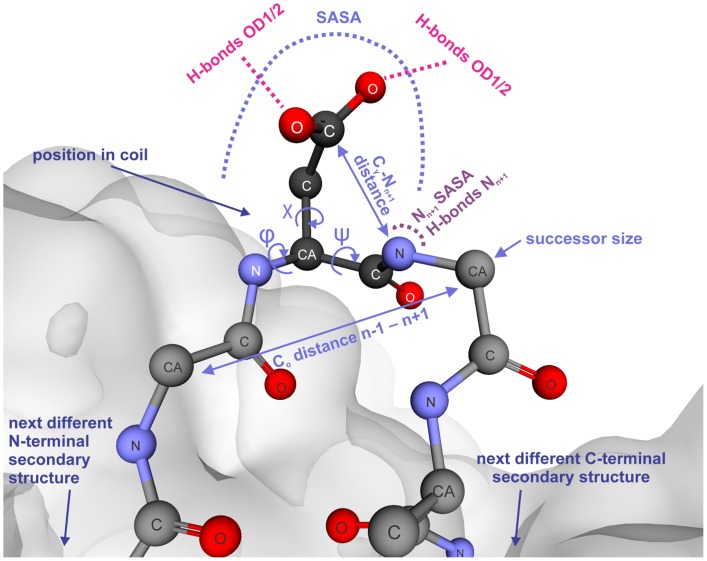
Parameters characterizing Asn and Asp residues in a structural environment outlined at an exemplary Asp residue. Parameters describing the carboxyl/amino group leaving tendency, the transition state accessibility, the N_n+1_ nucleophilicity, and the structural environment are depicted in pink, light blue, purple, and dark blue, respectively. Parameter names are used as in Table S1.

As the discrimination of both Asn/Asp degradation hotspots and stable Asn/Asp residues only based on primary sequence is prone to massive over-prediction [57], a set of 20 structural parameters described below was defined to reflect the 3D environment of these amino acids. They were chosen on the basis of their putative role in the degradation mechanism (Figure 1, Figure 3, Table S1) and were computationally extracted from the homology model ensembles.

A prerequisite for cyclic imide formation is the leaving tendency of the hydroxyl or the amino group of the Asp or Asn side chain, respectively. To estimate this tendency, the number of hydrogen bonds to the side chain oxygen atoms, or the side chain nitrogen atom was counted. For succinimide formation to occur, the carboxyl group of the Asp side chain must be protonated [39],[86]. The probable protonation state was obtained by calculating the structure-dependent Asp pK_a_ values using the PROPKA algorithm (SI Materials and Methods) [87]. Accessibility and high nucleophilicity of the succeeding backbone nitrogen are other potential prerequisites for succinimide formation (Figure 1). Therefore, the succeeding backbone nitrogen's solvent accessible surface area was calculated and the number of hydrogen bonds was counted.

The transition state of the succinimide formation reaction requires the Asp or Asn head group to approach the backbone nitrogen of the succeeding residue. Transition state-like conformation was probed by measuring the distance of the side chain C^γ^-atom to the N_n+1_-atom (Figs. 1, 3 [67]), the side chain dihedral angle χ_1_, and the dihedral angle CGONC that was defined as the angle between the atoms C^γ^, O, N_n+1,_ and C. Additionally, the solvent-accessible surface area of each Asp or Asn was calculated. It was shown that the residue succeeding an Asn or Asp influences the rate of succinimide formation [4], [6], [21], [34], [39], [57], [58], [60]. Hence, the successor amino acid size is recorded, as well as the backbone dihedral angles φ (C'_n-1_-N-C^α^-C') and ψ (N-C^α^-C'-N_n+1_) which provide information about the local structural conformation and thus the potential accessibility of the transition state.

Further parameters describe the broader structural environment. The root mean square deviation (RMSD) of the Asn/Asp residues' C^α^-atoms in the homology model ensemble reflects structural diversity within the ensemble and is seen as an indication of possible conformational flexibility. The secondary structure the residue is embedded in (helix, sheet, turn, or coil) [40], [68], and the distance to the next different N- and C-terminal secondary structure element [57] are included as additional parameters. If a residue is located in a coil secondary structure, its position within the coil (margin or center) was annotated (Materials and Methods). To quantify the “bend” of a coil tip, the distance between the C^α^-atoms of the n-1 and the n+1 residues was measured. Finally, the location within the F_v_ fragment (CDRs or framework) was attributed to each residue.

### Machine learning

Nine different machine learning methods were tested with the goal to find the optimal classifier for distinction between hotspots and non-hotspots in the F_v_ region. Our data set consisting of 185 models (37×5 models) contains in the case of Asn 55 hotspots and 940 non-hotspots, in the case of Asp 40 hotspots and 1425 non-hotspots, and was used for statistical analysis. Training of the classifiers was performed separately for Asn and Asp with a random 75% training dataset (always keeping the 5-membered ensembles together), excluding terminal residues as well as weak spots and reactive spots to avoid misleading classification.

**Figure 4:**
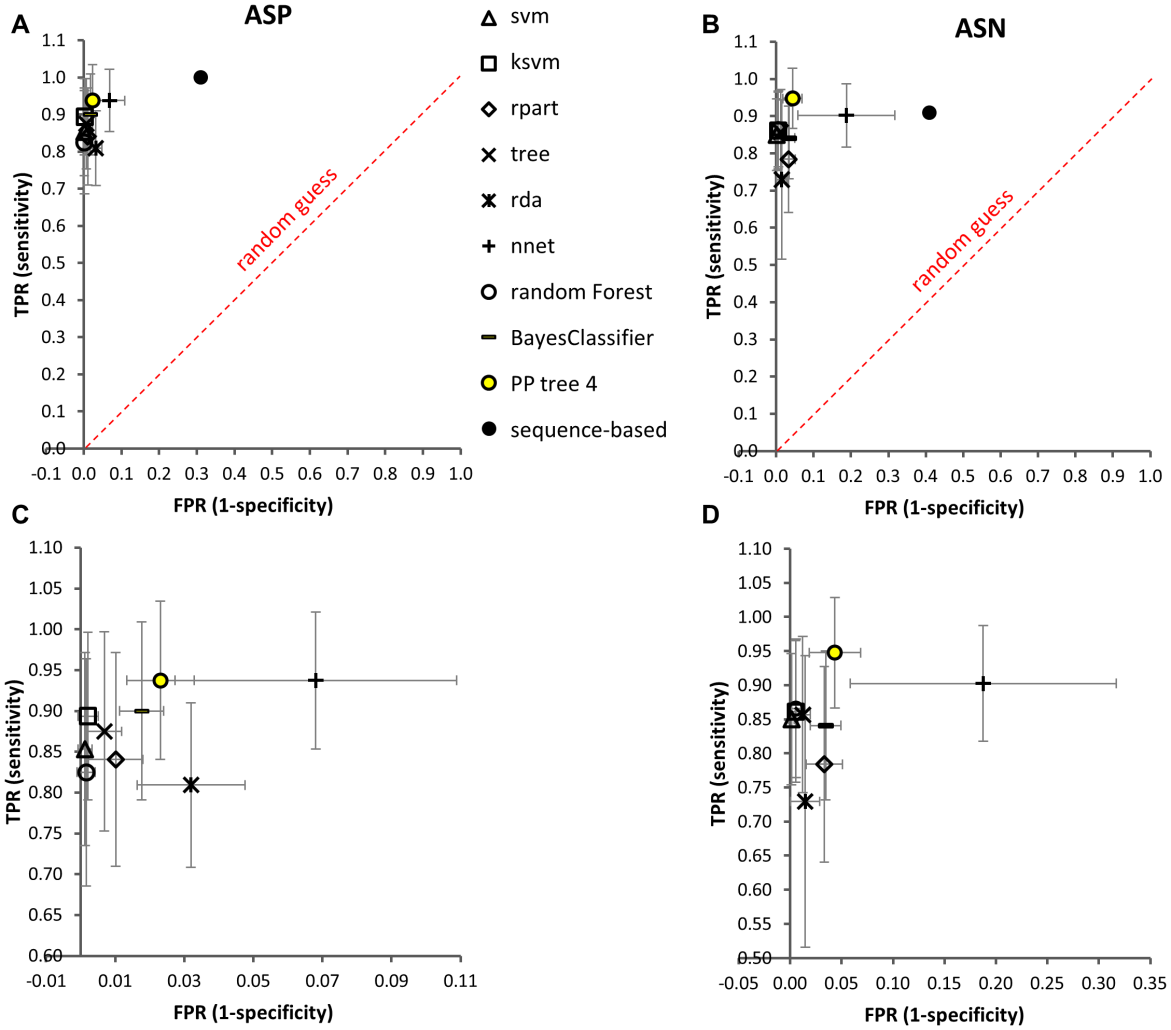
ROC plots for comparison of 3D classifiers to sequence-based prediction shows significant decrease of false-positive rates. Evaluation of different statistical methods is compared with only sequence-based prediction. For statistical classification methods, average numbers of false-positive and false-negative Asn/Asp residues are results of 40 rounds of Monte Carlo cross validation. TPR (true positive rate)  =  number of true positives divided by number of positives. FPR (false positive rate)  =  number of false positives divided by number of negatives. Tree, rpart, PP (Pipeline Pilot) tree, and RandomForest are recursive partitioning algorithms; svm, ksvm are support vector machine algorithms; rda is a regularized discriminant analysis algorithm; nnet is a neural network; sequence-based corresponds to prediction based on sequence motifs NG, NS, NT, and DG, DS, DT, DD, DH. The Pipeline Pilot tree, shown as a yellow circle, was selected as prediction algorithm, at pruning level 4. **A**: Asp model; **B**: Asn model. Panels **C** and **D** show a zoom view of the panels A and B, respectively. The numerical values shown in these graphs can be found in Table S3.

**Figure 5:**
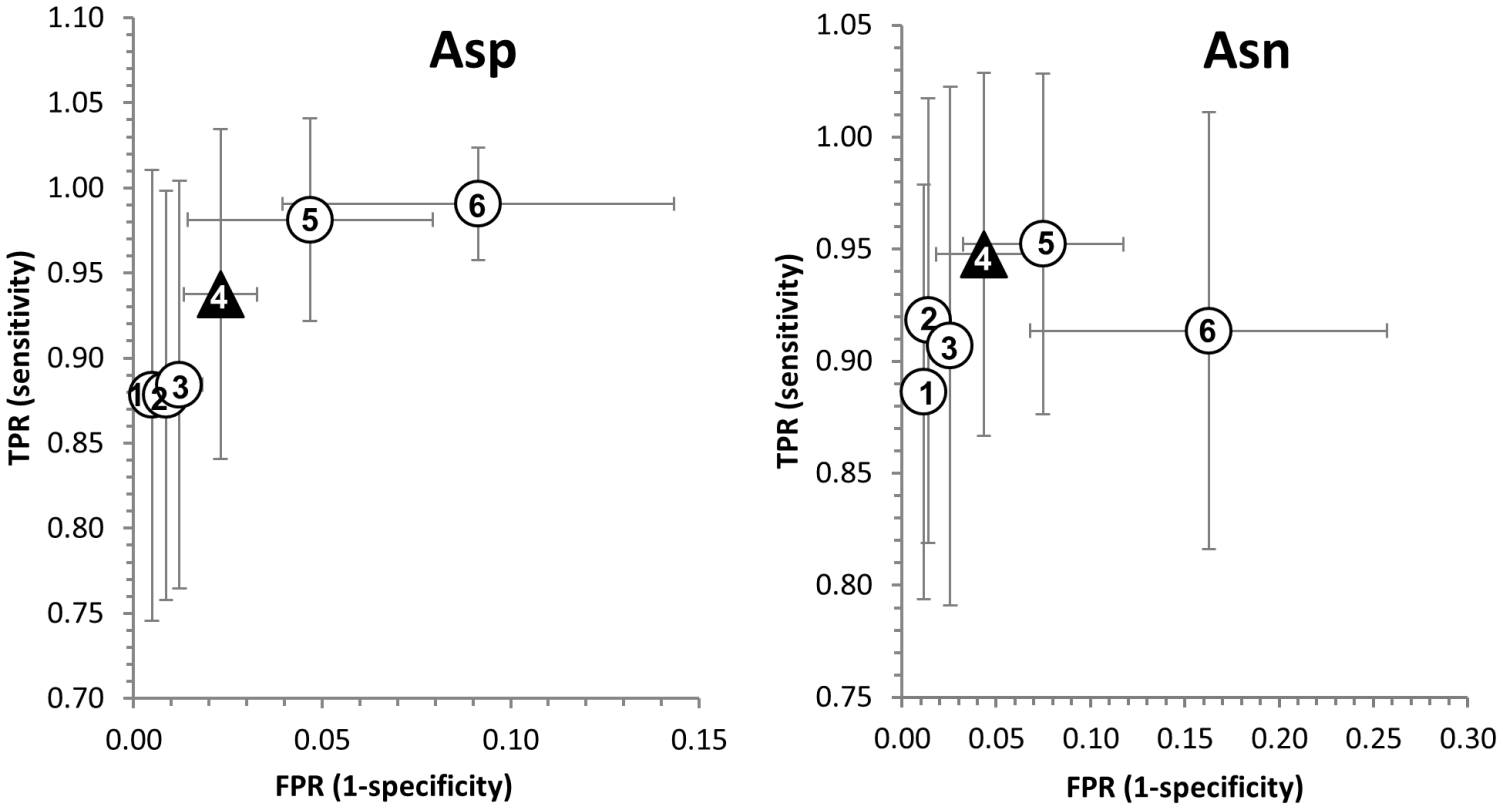
ROC plot for comparison of different pruning levels of decision trees. Decision trees were pruned automatically as implemented in Pipeline Pilot. Average numbers of false-positive and false-negative Asn/Asp residues are results of 40 rounds of Monte Carlo cross validation. TPR (true positive rate)  =  number of true positives divided by number of positives. FPR (false positive rate)  =  number of false positives divided by number of negatives. Trees 1-3 and 5-6 are shown as spheres, tree 4 as a black triangle. Tree 1 is the un-pruned tree model. Tree 4 was selected for prediction.

**Figure 6:**
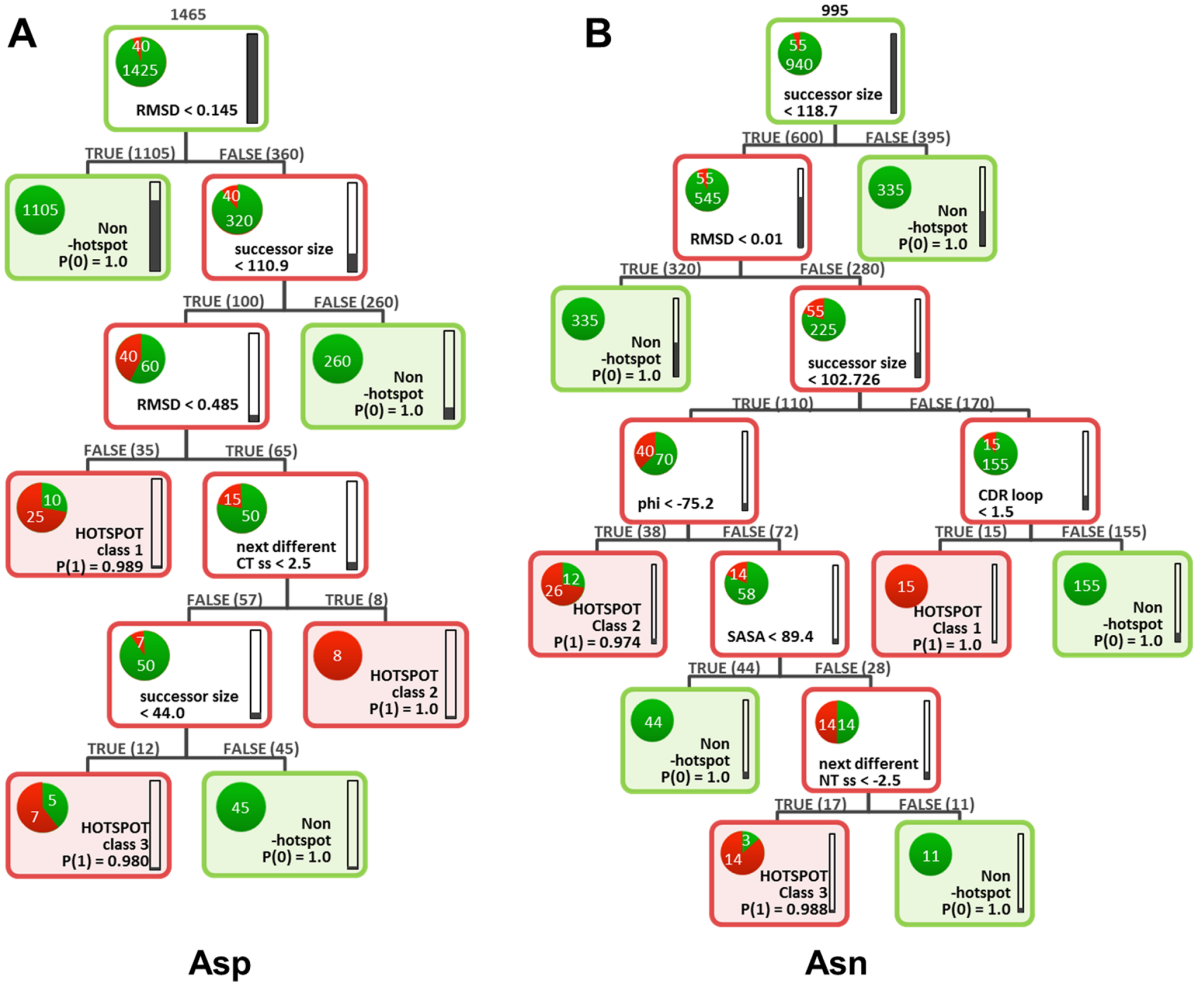
Final Aspartate (A) and asparagine (B) decision trees. The outline of nodes and leaves is colored by the weighted majority of the class that is present (red: hotspots, green: non-hotspots). Filling levels of the bars on the right hand side of each node/leaf refer to the fraction of the data set. The fraction of each class at a node/leaf is shown by the colored fraction of the circle. The number of members of each node/leaf is indicated above.

Bayesian classification, recursive partitioning, support vector machines, random forests, regularized discriminant analyses, and neuronal networks were tested in 40 repeats of random training set assignments (Monte Carlo cross validation), using all 20 parameters (SI Materials and Methods). Monte Carlo cross validation is described as a mathematically stringent validation approach in cases where no large, independent training and validation data sets are available [88], [89]. Asn and Asp classifications were separately dealt with because Asn degradation could follow different mechanisms [6], [42]–[45], (Figure 1), which led to an improved classification scheme. A residue counts as a predicted hotspot if at least one member of the five-membered ensemble was classified as such. To choose the optimal classifier out of the tested classification models, we used a receiver operating characteristic (ROC) analysis that is commonly applied to illustrate the performance of binary classification systems (SI Materials and Methods). Weighting a high true-positive rate as the most important criterion, the Pipeline Pilot implementation of a single-tree lookahead-enabled recursive partitioning algorithm [90] was chosen as the most suitable classifier and optimized for prediction purposes (Figure 4, Figure 5, SI Materials and Methods). The decision trees are shown in Figure 6.

After forty runs of test set validation against the model trained with randomized 75% training sets, an average of 0.5 out of 8 Asp-hotspots were not recognized, whereas an average of 6.6 out of 285 Asp non-hotspots were assigned false-positively. This corresponds to a TPR of 0.94, being the number of true positives (7.5) divided by the number of positives (8), and a FPR of 0.02, defined as the number of false positives (6.6) divided by the number of negatives (285) (Figure 4 A,C). In the case of Asn, an average of 0.6 out of 11 Asn-hotspots was assigned as false-negative (TPR = 0.95) and 8.1 out 188 non-hotspots were obtained as false-positives (FPR = 0.04) (Figure 4 B,D). This is a significant improvement to prediction based on solely primary sequence information, which led to a strong over-prediction in our dataset (Asp TPR = 1.0, FPR = 0.31; Asn TPR = 0.91, FPR = 0.41).

### Asp and Asn degradation propensity depends on residue flexibility, successor size, and secondary structure

In the case of Asp, the dataset consists of only 2.7% hotspots that need to be distinguished from the non-hotspot Asp residues. The first two decision tree splits can separate 93% of all non-hotspots (Figure 6 A). Non-hotspots are either inflexible or are succeeded by a large C-terminal amino acid. The remaining Asps to be classified show a high degree of conformational variability in the model ensembles and are succeeded by a small amino acid (Gly, Ala, Ser, Cys, or Asp). Of these, the first and largest Asp hotspot class is characterized by very high conformational variability (RMSD>0.485) and Asp, Cys, Ser, Ala or Gly as a successor. It contains 5 hotspots (5 members each) as well as 2 non-hotspot Asp residues (5 members each).

At the next node, hotspot class 2 is split off. Its 3 members (1 with 5 homology model members, 1 with 2, and 1 with 1 member only) are characterized by moderate conformational variability (RMSD between 0.145 and 0.485), are followed by either Asp, Cys, Ser, Ala or Gly, and show a change in C-terminal secondary structure within a stretch of less than 3 amino acids.

Hotspot class 3 represents an Asp-Gly motif with moderate conformational variability (RMSD 0.145–0.485) and a change in C-terminal secondary structure within more than 3 residues. It contains 2 hotspots (1 with 4 homology model members, and 1 with 3 members) and 1 false-positive Asp (5 members).

For Asn degradation hotspot classification, the main criteria are the size of the carboxy-terminal amino acid and conformational variability (Figure 6 B). Compared to the Asp dataset, there are twice as many Asn hotspots in relation to non-hotspots, which correspond to 5.5%. Also here, the first two decision tree splits can separate the bulk of non-hotspots (72%). Non-hotspots are succeeded by a big carboxy-terminal amino acid or are inflexible. The next split criterion is the successor size and leads to 2 branches, containing Asn residues with a successor size less or greater than 102.7 Å^2^. The latter is further categorized by the CDR loop location. Thus, the first Asn hotspot class contains residues in CDR loop 1, is characterized by carboxy-terminal residues such as Asp, Pro, Thr, or Asn, and is not inflexible (RMSD>0.01). It contains 3 hotspot members (5 homology model members each).

The residues with a successor size less than 102.7 Å^2^ are further classified by their backbone dihedral angle phi. Asn residues followed by Gly, Ala, Ser, or Cys (<102.7 Å^2^) that are not inflexible (RMSD>0.01) and whose phi angle is smaller than −75.2 degrees constitute the second and largest hotspot class 2. It contains 6 hotspot members (4 with 5 homology model members, 1 with 4, and 1 with 2 members), as well as 4 false-positives (1 with 5 homology model members, 2 with 3, and 1 with 1 member).

Hotspot class 3 is defined by the same flexibility and successor characteristics as class 2 but its 4 members (2 with 5 homology model members, 1 with 3, and 1 with 1 member only) feature a phi angle greater than −75.2 degrees, high solvent exposure (SASA>89.4 Å^2^) and a change in amino-terminal secondary structure within a stretch of more than 3 amino acids. Two non-hotspot Asn residues (1 and 2 homology model members) are also part of this class.

## Discussion

Spontaneous degradation of Asn and Asp residues in therapeutic proteins can occur during production, storage, and *in vivo*. In case of involvement in target binding, the formation of the degradation products succinimide, isoAsp, and Asp embedded in the CDRs can lead to loss of function or potency. The aim of this study was to gain insights into the structural basis of these degradation processes and thus allow for selection of chemically stable antibody variable domains.

Due to known limitations of sequence-based predictions of the propensity of Asn and Asp degradation, an *in silico* prediction tool was established to facilitate selection of stable antibody candidates. To this end we first obtained a uniform data set that contains residue-specific quantitative data on antibody degradation products. Where available, these detected modifications are in accordance with known hotspot information from published data [11], [12], [54], [91]. The pH was kept constant at 6.0 during forced degradation and sample preparation to detect the succinimide intermediate that quickly hydrolyzes at alkaline pH, Asp isomerization, which occurs mainly at slightly acidic pH, and Asn deamidation without method-induced deamidation events.

Usually, a mixture of Asp and iso-Asp is obtained in variable ratios after succinimide hydrolysis [4], [59], [63] which is the case for the majority of the deamidation events in our study. The occurrence of only one product in the published Asn degradation hotspot of Trastuzumab [11], which was shown to be Asp, supports a succinimide-independent degradation pathway – either via an alternative nucleophilic attack mechanism resulting in isoimide [42] or via direct Asn side chain hydrolysis [44] (Figure 1).

Several approaches to predict labile Asp and Asn residues from the sequence context or experimental X-ray structures were proposed [6], [16], [40], [42], [43], [45], [57], [67]–[70]. A tool for prediction of Asn deamidation but not Asp isomerization or succinimide formation in proteins was presented by Robinson & Robinson in 2001 [57]. The authors used reported deamidation rates of 198 Asn residues in 23 different proteins and 70 Asn residues in 61 human hemoglobin variants that were observed under a wide variety of experimental conditions. The main differences to our study are that (i) the prediction is only applicable for Asn, (ii) the hotspot collection – hence the basis for prediction – has a heterogeneous experimental background, (iii) the 3D information stems from experimental X-ray structures, not from homology models, (iv) for general users the prediction is possible for proteins with entries in the PDB until 2001, and (v) it requires an experimental structure for its application to new proteins. In comparison, the model proposed in our study is adapted to the variable region of therapeutic antibodies, and relies exclusively on *in silico* calculations, bypassing the need for experimental X-ray structures. The only prerequisites are (i) an antibody F_v_ domain sequence, (ii) a homology modeling tool, (iii) a molecular visualization software such as PyMol, and (iv), the statistical model presented in this work. The reduction of falsely assigned hotspots (average 2.3% for Asp, 4.3% for Asn) compared to sequence-only based prediction (31% for Asp, 43% for Asn) is reliable enough to employ this prediction during lead candidate selection. The cause for the described false-positives and also the false-negatives (6.3% Asp, 5.2% Asn) is the relatively small number of hotspots (8 Asp, 11 Asn) compared to non-hotspots (285 Asp, 188 Asn). Classification with only residues embedded in the CDR loop led to less predictive statistical values (not shown).

The best-performing predictor for this dataset is the Pipeline Pilot implementation of a lookahead-enabled single recursive partitioning tree. Partitioning trees provide split criteria in the order of their ability to split the dataset into hotspot and non-hotspot containing subsets. Thus, parameters utilized in the first nodes are those with a high discriminative power. Parameters which do not contribute to data splitting, either due to a lack of significance or due to noise, are omitted automatically.

The work presented here resulted in a tool to predict sites of antibody degradation and reveals the main characteristics that distinguish unstable and stable Asn and Asp amino acids in the variable region of mAbs: Asn and Asp residues with high flexibility and a small successor are prone to degradation. They can be further characterized by secondary structural elements. Interestingly, parameters most promptly describing the reaction mechanism (Figure 1) such as the distance between the C_
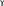
_ atom and backbone nitrogen atom of the C-terminal amino acid, the Asp pK_a_ value, or the side-chain dihedral angle χ_1_, were not relevant for classification.

The specificity of the prediction algorithm for antibodies can help to more efficiently pre-select mAbs in the process of finding the most stable, and simultaneously most potent clinical candidate molecule that is brought into further development, and into the clinic. By applying the algorithm, long-term and *in vivo* stability can be predicted, avoiding late stage failure. Filling the existing data set with more case studies representing the succinimide-independent Asn degradation pathway would probably further explain the structural prerequisites for this alternative mechanism. An adjustment to new molecule formats will be the next step in the future. With an expansion of the acquired knowledge to other protein classes, a broader application could be an interesting step ahead, providing a more general understanding of protein degradation mechanisms, independent of the protein class.

## Materials and Methods

### mAb origin

The marketed products used in this study include Avastin (Bevacizumab, Genentech/Roche); CYT387 (Nimotuzumab, Oncoscience, Ch.B.: 911017W002); Erbitux (Cetuximab, Bristol-Myers Squibb and Eli Lilly and Company, Lot: 7666001); Herceptin (Trastuzumab, RO-45-2317/000, Lot. HER401-4, Genentech); Humira (Adalimumab, Abbott, Ch.B.: 90054XD10); Prolia (Denosumab, Amgen, Ch.B.: 1021509); Raptiva (Efalizumab, Genentech, Merck Serono, Lot: Y11A6845); Remicade (Infliximab, Centocor, Ch.B.: 0RMA66104); Simulect (Basiliximab, Novartis, Ch.B.: S0014); Synagis (Pavilizumab, MedImmune, Lot.: 122-389-12); Tysabri (Natalizumab, Biogen Idec and Elan, LotA: 080475); Vectibix (Panitumumab, Amgen, Ch.B.: 1023731); and Xolair (Omalizumab, Genentech/Novartis, Ch.B.: S0053). The remaining 24 mAbs of the antibody collection stem from Roche and are human or humanized IgG1 or IgG4 antibodies.

### Generation of samples with induced degradation

All 37 therapeutic mAbs were subjected to induced degradation (stressed samples). To this end, 2 mg of each antibody were dialyzed over night at 4 °C into dilution buffer (20 mM histidine-chloride, pH 6.0) in D-Tube Dialyzers (Novagen, MWCO 6–8 kDa). Concentrations were determined by UV280 absorption and adjusted to 5 mg/ml with dilution buffer. After sterile filtration (Pall Nanosep MF, 0.2 µm) and transfer to sterile screw cap tubes, all mAb samples were quiescently incubated for 2 weeks at 40 °C.

### mAb sample preparation for tryptic peptide mapping experiments

80 µg of mAb reference and stressed sample were denatured and reduced for 1 h in a final volume of 124.5 µL of 100 mM Tris, 5.6 M guanidinium hydrochloride, 10 mM TCEP (tris(2-carboxyethyl)phosphine, Pierce Protein Biology Products, Thermo Fisher Scientific, Waltham, MA, USA), pH 6.0 at 37 °C. Buffer was exchanged to 20 mM histidine chloride, 0.5 mM TCEP, pH 6.0 in 0.5 ml Zeba Spin Desalting Columns (Pierce Protein Biology Products, Thermo Fisher Scientific, Waltham, MA, USA). mAbs were digested overnight at 37 °C by addition of 0.05 µg trypsin (Promega, Madison) per µg protein in a final volume of 140 µL. Digestion was stopped by addition of 7 µL of 10% formic acid (FA) solution, and samples were frozen at −80°C until further analysis.

### Detection of modified peptides by liquid-chromatography tandem mass-spectrometry

14 µg of digested protein were applied to an RP-HPLC (Agilent 1100 Cap LC, Agilent Technologies, Böblingen, Germany) on a Varian Polaris 3 C18 – Ether column (1×250 mm; 3 µm particle diameter, 180 Å pore size) from Varian (Darmstadt, Germany) for separation. The mAb2, mAb14, and Nimotuzumab digest were additionally separated by RP-UPLC (ACQUITY BEH300 C18 column, 1×150 mm, 1.7 µm bead size, 300 Å pore size, Waters, Manchester, UK). The HPLC or UPLC eluate was split using Triversa NanoMate (Advion, Ithaca, NY, USA) and 380 nl/min were infused into a LTQ Orbitrap classic tandem mass spectrometer (Thermo Fisher Scientific, Waltham, MA, USA) operating in positive ion mode. The mobile phases of RP-HPLC consisted of 0.1% FA in water (solvent A) and 0.1% FA in acetonitrile (solvent B). The HPLC was carried out using a stepwise gradient starting at 2% solvent B, elevated to 15% from min 5-15, to 32% from min 15-70, to 38% from min 70-80, to 100% from min 80-90, and finally dropped to 2% from min 92–110 with a flow rate of 60 µL/min. UPLC was effected with a linear gradient from 1 to 40% solvent B from 0 to 130 min. UV absorption was measured at wavelengths of 220 and 280 nm. Data acquisition was controlled by Xcalibur software (Thermo Fisher Scientific, Waltham, MA, USA). Parameters for MS detection were adjusted according to general experience available from peptide analyses of recombinant antibodies. For MS/MS measurements, fragmentation was induced by low-energy CID using helium as a collision gas with 35% collision energy in the LTQ. To obtain higher resolution of the fragment ions for mAb14 and Nimotuzumab, the fragmentation was performed in the Orbitrap using a parent mass list, an isolation width of 3, a parent mass width of 0.2 Da, AGC Target 400000, and acquisition time of 5000 ms.

### mAb14 and Nimotuzumab sample preparation for MS/MS evaluation

For further characterization, mAb14 and Nimotuzumab stressed samples were treated as follows. 250 µg of mAb was denatured by addition of denaturing buffer (0.4 M Tris (Sigma-Aldrich, Taufkirchen, Germany), 8 M guanidinium hydrochloride (Sigma-Aldrich, Taufkirchen, Germany), pH 8) to a final volume of 240 µL. Reduction was achieved by addition of 20 µL of 0.24 M dithiothreitol (DTT) (Roche, Mannheim, Germany) freshly prepared in denaturing buffer and incubation at 37 °C for 60 min. Subsequently, the sample was alkylated by addition of 20 µL of 0.6 M iodoacetic acid (Merck, Darmstadt, Germany) in water for 15 min at room temperature in the dark. The excess of alkylation reagent was inactivated by addition of 30 µL of DTT solution. The samples were then buffer exchanged to approximately 480 µL of 50 mM Tris/HCl, pH 7.5 using NAP5 Sephadex G-25 DNA grade columns (GE Healthcare, Germany). The mAbs were digested 5 h at 37 °C by addition of 0.03 µg trypsin (Promega, Madison) per µg protein in a final volume of 500 µL. Digestion was stopped by addition of 20 µL of 10% formic acid (FA)-solution, and samples were frozen at −80°C until further analysis.

### Data analysis for the quantification of modification levels

SIEVE software version 2.0 (VAST Scientific Inc., Cambridge, MA) was used to pre-filter data for differences between stressed and reference samples. Crucial SIEVE settings were a frame time width of 1.0 min, *m/z* width of 8.0 ppm, and an intensity threshold of 50000 counts. SIEVE data filtered for monoisotopic masses (prelement = 0) was imported into a macro-enabled Excel workbook as well as data from *in silico* tryptic digestion of mAbs' heavy and light chains, containing theoretical mass-to-charge ratios of modified and unmodified peptides (in-house data processing software). Differences in signal intensities or retention time (reference vs. stress) of relevant *m/z* values of peptides were detected in a semi-automatized fashion by a macro-enabled EXCEL workbook (Microsoft, Redmond, WA, USA). The resulting pre-filtered peptides from 76 peptide maps were manually inspected to verify Asn and Asp modifications by their *m/z*-values within the experimental mass spectrum. For quantification, extracted ion chromatograms (XICs) of peptides of interest were generated on the basis of their monoisotopic mass and detected charge states using Xcalibur Software (Thermo Fisher Scientific, Waltham, MA, USA). Relative amounts of modified vs. unmodified peptides were calculated after manual integration of the corresponding peak areas. Additionally, all peptides lying in the CDR regions containing a putative hotspot motif (Asn-Gly, Asn-Thr, Asn-Ser, Asn-Asn, Asp-Gly, Asp-Thr, Asp-Ser, Asp-Asp, Asp-His) were analyzed even if not alerted after SIEVE software analysis to ensure completeness of the data.

### Homology modeling and extraction of 2 and 3-dimensional parameters

Homology models were built with an automated software script for the program MODELER 9v7 [92]. Modeling templates were chosen based on sequence conservation from a reference structure database consisting of human, mouse, and chimeric antibody Fab fragment crystal structures with a minimum resolution of 2.8 Å, and without missing internal residues in their variable regions. The best resulting model for each mAb was used as a basis for a loop refinement procedure (LOOPER, [85]) [93]. In turn, the 5 most likely solutions from loop refinement were selected and used as an ensemble of structures for each mAb. Parameters were extracted computationally from these homology model ensembles (Table S1). The pK_a_ value was calculated using the program PROPKA as part of pdb2pqr [87]. The secondary structure elements (sheet, helix, turn, coil) were extracted with a custom script using Discovery Studio [85]. The parameters “next different N-terminal secondary structure”, “next different C-terminal secondary structure” and “position in coil” were deduced from the secondary structure information of surrounding residues using Boolean rules (Table S1) implemented in Pipeline Pilot [90]. A “margin” “position in coil” is assigned if the next different secondary structure element is one or two residues away, either in N- or C-terminal direction. A “center” “position in coil” is assigned if in both N- and C-terminal direction the secondary structure is the same for 4 residues or in both directions for more than 4 residues. The parameter “F_ab_ location” is a number that was deduced from combined Chothia and Kabath CDR definitions for antibodies [94]. “F_ab_ location” number 1 corresponds to framework 1 of the heavy chain (FR H1), 2 to CDR H 1, 3 to FR H 2, 4 to CDR H 2, 5 to FR H 3, 6 to CDR H 3, 7 to FR H 4, 8 to framework 1 of the light chain (FR L1), 9 to CDR L 1, 10 to FR L 2, 11 to CDR L 2, 12 to FR L 3, 13 to CDR L 3, and 14 to FR L 4. “CDR loop” is a number ranging from 1 to 3, equal for light and heavy chain. “Successor size” is the solvent accessible surface area of a fully exposed amino acid[95] in Å^2^ and is defined as follows: Ala, 64.78; Cys, 95.24; Asp, 110.21; Glu, 143.92; Phe, 186.7; Gly, 23.13; His, 146.45; Ile, 151.24; Lys, 177.37; Leu, 139.52; Met, 164.67; Asn, 113.19; Pro, 111.53; Gln, 147.86; Arg, 210.02; Ser, 81.22; Thr, 111.6; Val, 124.24; Trp, 229.62; Tyr, 200.31. Terminal residues (lacking phi and psi) are marked in our data collection. All other parameters were extracted from the PDB files with self-written python scripts in PyMOL [96](Table S1).

### Machine learning algorithms used for classification assessment

In order to find the best possible classifier, several different binary classification methods that appeared suitable for this type of classification problem, were tested, namely support vector machines, recursive partitioning algorithms, regularized discriminant analysis and neuronal networks. They were available as packages for the statistical software R or in Pipeline Pilot [90]. Support vector machines (SVM) offer different ways to transform a given data set into higher dimensions with the help of a so called kernel function. Here, the svm method [97] from the package e1071 and the ksvm method from the kernlab package [98] were used. Recursive partitioning methods identify parameters in a step-wise manner to split the given data set into subsets, thereby producing a decision tree. The difference between the algorithms is mainly based on different methods to decide on the best splitting parameter in a given step. The “tree” [99] and “rpart” [100] methods were used in R whereby several different splitting methods were tested, as well as the recursive partitioning tree implementation in Pipeline Pilot.

A more generalized form of classifier can be achieved by combining decision trees based upon subsets of the original training set into a so-called random forest. Regularized discriminant analysis builds a classifier by combining a subset of the available parameters using regularized group covariance matrices in order to achieve best possible discrimination. This method is implemented as the function “rda” in the klaR package [101]. A neural network tries to emulate the basic functionality of one or several interconnected layers of neurons. A so-called single-hidden-layer neural network as implemented in the “nnet” method of R [102] was applied. Finally, a naïve Bayes classifier, a probabilistic method that uses Bayes' theorem to compute probabilities of a data sample belonging to a certain class, given the training data, was tested as implemented in the “NaiveBayes” method of R.

As a highly imbalanced dataset with very few hotspots but many non-hotspots had to be dealt with, class weights were introduced to put more emphasis on the minority class. A standard weighting scheme was identified, using the inverse of the class frequency, as the best in terms of classification error with special emphasis on the false negative rate.

### Classification evaluation

We used a receiver operating characteristic (ROC) analysis that is commonly applied to illustrate the performance of binary classification systems. Hereby, the fraction of true positives out of the positives (true positive rate, TPR) is plotted against the fraction of false positives out of the negatives (false positive rate, FPR). Weighting a high true-positive rate as the most important criterion, the Pipeline Pilot implementation of a single-tree lookahead-enabled recursive partitioning algorithm [90] was chosen as the most suitable classifier (Figure 4). At each step, the recursive partitioning algorithm determines a parameter and a threshold value that is the best in splitting the dataset into homogeneous subsets belonging to one class (hotspot or non-hotspot). The splitting point is called a node, and the class is called a leaf. The integrated lookahead functionality ensures that the chosen splitting parameter and threshold value is not only optimal for the given step but also for subsequent steps. Like this, the model identifies the most crucial parameters for distinguishing hotspots from non-hotspots. This classifier yields the best combination of a high TPR, a low FPR for prediction of Asn and Asp degradation propensity, and good algorithm interpretability, even after the following optimization procedure for prediction purposes.

The Asn and Asp single-tree lookahead-enabled recursive partitioning algorithms were optimized in order to enhance model performance for new data and to avoid over-fitting. Therefore, Asn and Asp trees were pruned, i.e. branches were systematically removed to yield smaller trees. To test the pruned models' predictivity, they were validated against a 25% test set in forty independent runs (Figure 5). Final Asn and Asp algorithms were trained with 100% of the data, and were chosen on the basis of the corresponding ROC plots (Figure 4) as well as meaningful tree interpretability. A lookahead depth of 4 with 7 lookahead alternatives and pruning level 4 were used. They are represented as decision trees in Figure 6.

### Recursive partitioning and prediction

Terminal residues as well as residues with less than 3% modification rate in the stressed sample (weak spots and reactive spots) were excluded from the training. All 20 parameters described were supplied to the training set. A main feature of the single-tree recursive partitioning classification algorithm in Pipeline Pilot is the opportunity to assign a certain “look-ahead” depth that allows for better classification due to testing more alternative splits.

The two resulting prediction models are applied to new data. The programmed rule for a hotspot alert is the following: if at least one Asn/Asp in a set of five homology models is predicted to be a hotspot, the residue per se is classified as such. The probability for hotspot classification can range from a 0.5 minimum to a 1.0 maximum for each member of the ensemble. Thus, prediction output is not only qualitative but also quantitative, expressed in the average of the probabilities of each member for being a hotspot including the standard deviation. Like this, the information if one, two, three, four, or five members of the ensemble are in hotspot conformation, is contained in the prediction output.

## Supporting Information

Figure S1 **Example of extracted ion current chromatograms and tandem mass spectra for detection and localization of a deamidated peptide.** **A**. Extracted ion current chromatograms of the unmodified tryptic peptide SINSATHYAESVK at *m/z* 703.84 and 469.56 (charges 2+ and 3+, upper panel) and its deamidated form at *m/z* 704.34 and 469.89 (charges 2+ and 3+, lower panel). Deamidation corresponds to a mass increase of 0.98 Da. The unmodified peptide elutes at 18.4 min. The deamidated species (peak 1 and 2) are eluting at 18.7, and 19.2 min **B**. y^+^ fragment ions of the deamidated peptide SINSATHYAESVK and their theoretical masses (*m/z*) **C**. MS2 spectrum of the unmodified peptide at *m/z* 703.84 (charge 2+) and the deamidated peptide at *m/z* 704.34 (charge 2+). The y^+^ fragment ions supporting the deamidation of the Asn-Ser motif compared to the unmodified peptide were detected in the LTQ **D**. Zoom into y fragment ions 10 and 11. The deamidation-induced y_10_^+^ shift is indicated by a red arrow.
(TIF)

Figure S2 **Conformational flexibility of loops is captured by use of 5 homology modeling solutions.** The 5 most likely solutions of the loop refinement procedure are structurally superimposed. For illustration purposes, only the side chains of the CDR H3 are shown as lines and in different colors per model.
(TIF)

Table S1 **Weak spots which were excluded from the training dataset because the extent of modification (>1.0 and <3.0% after stress) is detectable but considered irrelevant for stability under real-time storage conditions.**
(DOCX)

Table S2 **Reactive spots which were excluded from the training dataset because the extent of modification (<1.0% after stress) is detectable but considered irrelevant for stability under real-time storage conditions.**
(DOCX)

Table S3 **Comparison of the various classifiers.** Abbreviations and data origins are analogous to Figure 4.
(DOCX)
