# Supplementary figures and images for: Structure-Based Prediction of Asparagine and Aspartate Degradation Sites in Antibody Variable Regions
Source: PLoS One. 2014 Jun 24;9(6):e100736. doi: 10.1371/journal.pone.0100736 (PMC4069079; doi:10.1371/journal.pone.0100736)

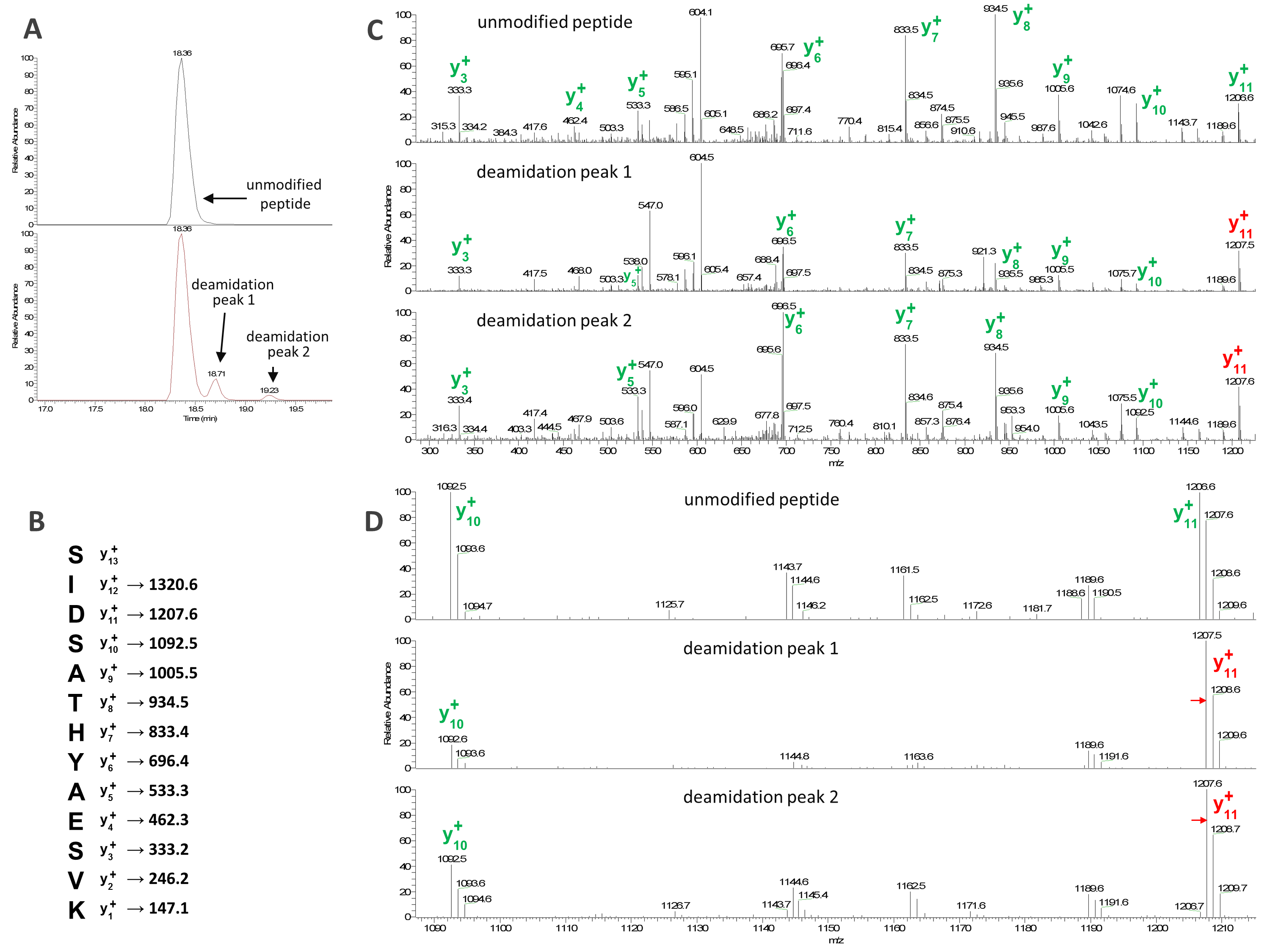

Supplement: Figure S1 — Example of extracted ion current chromatograms and tandem mass spectra for detection and localization of a deamidated peptide. A. Extracted ion current chromatograms of the unmodified tryptic peptide SINSATHYAESVK at m/z 703.84 and 469.56 (charges 2+ and 3+, upper panel) and its deamidated form at m/z 704.34 and 469.89 (charges 2+ and 3+, lower panel). Deamidation corresponds to a mass increase of 0.98 Da. The unmodified peptide elutes at 18.4 min. The deamidated species (peak 1 and 2) are eluting at 18.7, and 19.2 min B. y+ fragment ions of the deamidated peptide SINSATHYAESVK and their theoretical masses (m/z) C. MS2 spectrum of the unmodified peptide at m/z 703.84 (charge 2+) and the deamidated peptide at m/z 704.34 (charge 2+). The y+ fragment ions supporting the deamidation of the Asn-Ser motif compared to the unmodified peptide were detected in the LTQ D. Zoom into y fragment ions 10 and 11. The deamidation-induced y10 + shift is indicated by a red arrow. (TIF) [file pone.0100736.s001.tif]

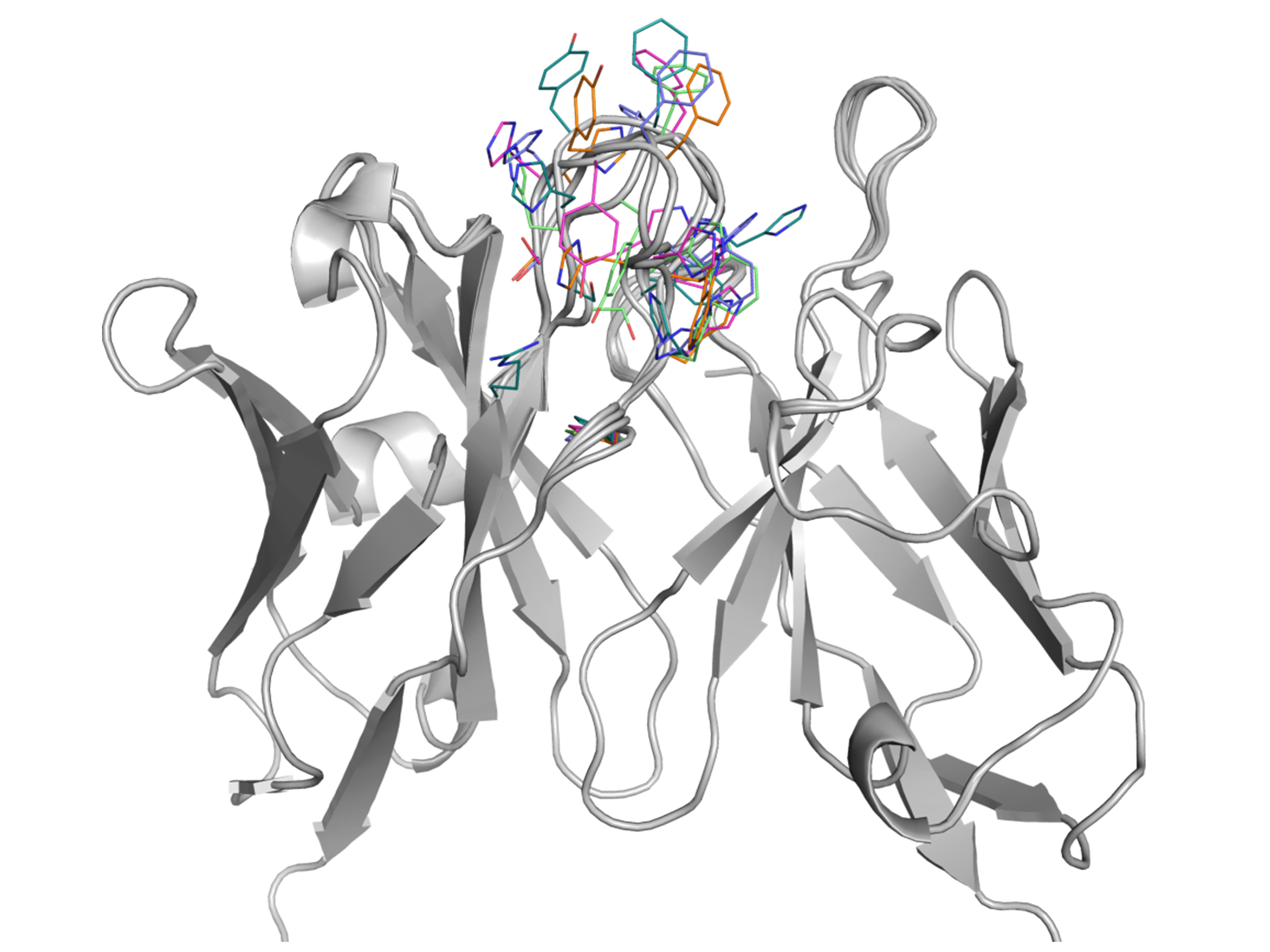

Supplement: Figure S2 — Conformational flexibility of loops is captured by use of 5 homology modeling solutions. The 5 most likely solutions of the loop refinement procedure are structurally superimposed. For illustration purposes, only the side chains of the CDR H3 are shown as lines and in different colors per model. (TIF) [file pone.0100736.s002.tif]
