# Supplementary material for: Structure-Based Prediction of Asparagine and Aspartate Degradation Sites in Antibody Variable Regions
Source: PLoS One. 2014 Jun 24;9(6):e100736. doi: 10.1371/journal.pone.0100736 (PMC4069079; doi:10.1371/journal.pone.0100736)
Supplement: Table S1 — Weak spots which were excluded from the training dataset because the extent of modification (>1.0 and <3.0% after stress) is detectable but considered irrelevant for stability under real-time storage conditions. (DOCX) [file pone.0100736.s003.docx]

| **mAb** | **modification** | **% modified (stressed)** | **motif** | **location** |
| --- | --- | --- | --- | --- |
| mAb14 | suc | 2.9 | ?^#^ | LC CDR 1 |
| mAb2 | iD | 2.8 | DS | LC CDR 2 |
| Trastuzumab | suc | 2.8 | NG | HC CDR 2 |
| Trastuzumab | suc | 2.3 | DG | HC CDR 3 |
| mAb16 | suc | 2.2 | NT | HC CDR 2 |
| mAb33 | suc | 2.0 | DD or DA | HC CDR 3 |
| Trastuzumab | dea | 2.0 | NG | HC CDR 2 |
| mAb12 | suc | 1.9 | DG | HC CDR 2 |
| mAb11 | suc | 1.8 | DG | HC CDR 2 |
| mAb16 | suc | 1.8 | DD | HC CDR 2 |
| Natalizumab | dea | 1.4 | NG | HC CDR 2 |
| mAb2 | suc | 1.3 | NN | LC CDR 1 |
| mAb1 | iD+suc | 1.0 | DS | HC CDR 2 |
| mAb2 | suc | 1.0 | DS | LC CDR 3 |

^#^proof of modification site impossible with available methods
