# Supplementary material for: Structure-Based Prediction of Asparagine and Aspartate Degradation Sites in Antibody Variable Regions
Source: PLoS One. 2014 Jun 24;9(6):e100736. doi: 10.1371/journal.pone.0100736 (PMC4069079; doi:10.1371/journal.pone.0100736)
Supplement: Table S2 — Reactive spots which were excluded from the training dataset because the extent of modification (<1.0% after stress) is detectable but considered irrelevant for stability under real-time storage conditions. (DOCX) [file pone.0100736.s004.docx]

| **mAb** | **modification** | **% modified (stressed)** | **motif** | **location** |
| --- | --- | --- | --- | --- |
| Adalimumab | suc | < 1.0 | DD | HC CDR 1 |
| Adalimumab | suc | < 1.0 | NS | HC CDR 2 |
| Cetuximab | dea | < 1.0 | NS | HC FW3 |
| Efalizumab | suc | < 1.0 | DS | HC CDR 2 |
| mAb1 | suc | < 1.0 | NT | HC CDR 3 |
| mAb1 | suc | < 1.0 | DS | HC CDR 2 |
| mAb10 | suc | < 1.0 | DS | HC CDR 2 |
| mAb12 | suc | < 1.0 | DS | LC CDR 2 |
| mAb12 | iD+suc | < 1.0 | DS | LC CDR 1 |
| mAb16 | suc | < 1.0 | DT | LC CDR 2 |
| mAb16 | suc | < 1.0 | NN | LC CDR 2 |
| mAb17 | suc | < 1.0 | NS | LC CDR 1 |
| mAb18 | suc | < 1.0 | DA | LC CDR 2 |
| mAb27 | suc | < 1.0 | NT | LC CDR 3 |
| mAb28 | suc | < 1.0 | NS | HC CDR 1 |
| mAb29 | suc | < 1.0 | DA | LC CDR 2 |
| mAb3 | suc | < 1.0 | DA | HC CDR 3 |
| mAb32 | suc | < 1.0 | DS | HC CDR 3 |
| mAb33 | suc | < 1.0 | ? ^#^ | HC CDR 2 |
| mAb9 | suc | < 1.0 | DT | LC CDR 2 |
| Nimotuzumab | suc | < 1.0 | DS | HC CDR 3 |
| Omalizumab | suc | < 1.0 | DG | HC CDR 2 |
| Pavilizumab | suc | < 1.0 | ? ^#^ | HC CDR 2 |
| Pavilizumab | suc | < 1.0 | DT | LC CDR 2 |

^#^proof of modification site impossible with available methods
