# Supplementary material for: Structure-Based Prediction of Asparagine and Aspartate Degradation Sites in Antibody Variable Regions
Source: PLoS One. 2014 Jun 24;9(6):e100736. doi: 10.1371/journal.pone.0100736 (PMC4069079; doi:10.1371/journal.pone.0100736)
Supplement: Table S3 — Comparison of the various classifiers. Abbreviations and data origins are analogous to Figure 4. (DOCX) [file pone.0100736.s005.docx]

| Asp Classifier | FPR | STDEV (FPR) | TPR | STDEV (TPR) |
| --- | --- | --- | --- | --- |
| svm | 0.001 | 0.002 | 0.85 | 0.12 |
| ksvm | 0.002 | 0.003 | 0.89 | 0.10 |
| rpart | 0.010 | 0.008 | 0.84 | 0.13 |
| tree | 0.007 | 0.005 | 0.88 | 0.12 |
| rda | 0.032 | 0.016 | 0.81 | 0.10 |
| nnet | 0.068 | 0.041 | 0.94 | 0.08 |
| randomForest | 0.002 | 0.003 | 0.83 | 0.14 |
| BayesClassifier | 0.018 | 0.006 | 0.90 | 0.11 |
| PP tree 4 | 0.023 | 0.010 | 0.94 | 0.10 |
| Sequence-based | 0.310 | - | 1.00 | - |

| Asn Classifier | FPR | STDEV (FPR) | TPR | STDEV (TPR) |
| --- | --- | --- | --- | --- |
| svm | 0.001 | 0.003 | 0.85 | 0.10 |
| ksvm | 0.005 | 0.005 | 0.86 | 0.10 |
| rpart | 0.033 | 0.018 | 0.78 | 0.14 |
| tree | 0.012 | 0.007 | 0.86 | 0.11 |
| rda | 0.015 | 0.014 | 0.73 | 0.21 |
| nnet | 0.188 | 0.129 | 0.90 | 0.08 |
| randomForest | 0.006 | 0.006 | 0.87 | 0.10 |
| BayesClassifier | 0.035 | 0.015 | 0.84 | 0.11 |
| PP tree 4 | 0.043 | 0.025 | 0.95 | 0.08 |
| sequence based | 0.410 | - | 1.00 | - |
